# Ribosomal RNA Pseudouridylation: Will Newly Available Methods Finally Define the Contribution of This Modification to Human Ribosome Plasticity?

**DOI:** 10.3389/fgene.2022.920987

**Published:** 2022-06-01

**Authors:** Chiara Barozzi, Federico Zacchini, Sidra Asghar, Lorenzo Montanaro

**Affiliations:** ^1^ Dipartimento di Medicina Specialistica, Diagnostica e Sperimentale (DIMES), Alma Mater Studiorum—Università di Bologna, Bologna, Italy; ^2^ Centro di Ricerca Biomedica Applicata, CRBA, Universita di Bologna, Policlinico di Sant’Orsola, Bologna, Italy; ^3^ Departmental Program in Laboratory Medicine, IRCCS Azienda Ospedaliero-Universitaria di Bologna, Bologna, Italy

**Keywords:** rRNA, ribosome, pseudouridine detection, cancer, snoRNA

## Abstract

In human rRNA, at least 104 specific uridine residues are modified to pseudouridine. Many of these pseudouridylation sites are located within functionally important ribosomal domains and can influence ribosomal functional features. Until recently, available methods failed to reliably quantify the level of modification at each specific rRNA site. Therefore, information obtained so far only partially explained the degree of regulation of pseudouridylation in different physiological and pathological conditions. In this focused review, we provide a summary of the methods that are now available for the study of rRNA pseudouridylation, discussing the perspectives that newly developed approaches are offering.

## Introduction

Ribosome biogenesis is a highly complex process that requires the accomplishment of different steps. One of these fundamental steps is represented by the modification of ribosomal RNA (rRNA) at specific sites. These modifications are required for the proper maturation and folding of rRNA and impact its stability (Liang et al., 2009; Abou Assi et al., 2020). It is also known that the modifications can play a relevant role in the catalytic activity of the ribosome (King et al., 2003). The two most abundant modifications in rRNA are represented by ribose 2′-O-methylation and the isomerization of uridines to pseudouridines (often abbreviated with the letter Ψ). In particular, more than 100 rRNA uridines are known to be targets of pseudouridylation. Most of these modification sites are clustered close to functionally important regions of the ribosome (Decatur and Fournier, 2002; Natchiar et al., 2017). Some of these sites are universally conserved, while others are eukaryotic- or human-specific (Natchiar et al., 2017). The presence of pseudouridine confers greater conformational stability to the rRNA, a feature necessary for proper rRNA folding and interaction with ribosomal proteins (Sumita et al., 2005). Changes in the level of rRNA uridine modification can alter ribosome structure and ultimately its activity (King et al., 2003; Liang et al., 2007), affecting translational fidelity and sensitivity to ribosome-based antibiotics (Baxter-Roshek et al., 2007; Liang et al., 2007; Piekna-Przybylska et al., 2008). Moreover, accumulating evidence suggests pseudouridylation plays a major role in modulating the interactions between tRNA, rRNA and mRNA thereby affecting translation (Jack et al., 2011; Bastide and David, 2018). In particular, it was recently demonstrated that the presence of pseudouridine in mRNA hampers translation elongation and increases the occurrence of amino acid substitutions, supporting the idea that these mRNA modifications can modulate mRNA translatability and providing evidence that pseudouridine can alter tRNA selection by the ribosome (Eyler et al., 2019). It is increasingly more evident that RNA interactions with its several heterogenous modifications are at the heart of ribosome function and acquiring the molecular details of these interactions is a challenge in order to comprehend how the ribosome fine-tunes gene expression control in physiological and pathological conditions.

Alterations of pseudouridylation in rRNA are also known to play an active role in the development of human disorders such as cancer (Ruggero et al., 2003; Montanaro et al., 2006) and a rare inherited multisystemic syndrome termed X-linked dyskeratosis congenita, due to mutations of the gene encoding for dyskerin (Heiss et al., 1998), the pseudouridine synthase acting on rRNA. Specifically, there is evidence that alterations in ribosome functions caused by changes in pseudouridylation levels of rRNA may contribute to tumor development by modifying the translation of key cancer genes (Montanaro, 2010).

Despite its clear role in enabling proper ribosome function and its involvement in human pathology, the mechanism underlying the contribution of rRNA pseudouridylation in the development of human disorders remained underexplored for a long time. This relatively limited knowledge may be considered the consequence of the lack of proper reliable technical approaches. In fact, site-specific quantification of pseudouridine on a wide scale represented a major technical issue for a long time, but recent developments in high throughput sequencing approaches enabled us to obtain increasingly precise and reliable quantifications on a transcriptomic scale.

This review aims to give an updated perspective of the available technical approaches that can be employed to fully characterize the role of altered rRNA pseudouridylation in the development of different human pathologies.

## Pseudouridylation Detection Methods

Until recently, the study of pseudouridylation was hampered by biological and technical issues, because the isomeric form of pseudouridine possesses no distinguishable features nor mass changes. This limited the availability of methods for site-specific detection of pseudouridine, required a previous knowledge of the specific position of the pseudouridine in the sequence analyzed, and was suitable only for highly represented transcripts.

Over the last few years, different approaches were developed for the quantitative and site-specific identification of the pseudouridine landscape in cellular models and tissue samples. They may be low throughput, for the identification or validation of specific pseudouridine sites, and high throughput for an overview of all sites of modification in a specific class of RNA or the whole transcriptome.

The major features of the techniques reported for the detection of pseudouridines are summarized in Supplementary Table S1. For each technique, it provides the concept on which the method relies on, the main advantages and disadvantages, the scale and the RNA class of application with the relevant references.

### Low Throughput Methods for Pseudouridine Detection in RNA

Direct chemical analysis of RNA post-transcriptional modifications has been performed extensively by liquid chromatography followed by mass spectrometry (LC-MS) (Durairaj and Limbach, 2008; Thüring et al., 2016). Since pseudouridylation is a mass-silent modification, conventional MS-based analysis relies on direct chemical labeling of pseudouridine (Ψ) on RNA (with cyanoethylation or acrylonitrile) or the metabolic pre-labeling of RNA with 5,6-D2-uracil. However, protocols for chemical labeling of pseudouridine are generally laborious and can suffer from limited selectivity (Kellersberger et al., 2004). Only recently, with the use of CRISPR-Cas9 technology and direct nanoflow LC-MS, the *in vivo* deuterium labeling was successfully applied for direct determination of Ψs in cellular non coding RNA as rRNA and small nuclear RNA (and in such case at subpicomole amounts) (Yamaki et al., 2020). This method allowed also the stoichiometric quantification of the modifications at each pseudouridylation site without the need of synthetic reference RNA (as by the SILNA method, described below). However, this approach, although particularly sensitive and specific, requires a complex cellular system and instrumentations, thus limiting this application. Pseudouridine was hence distinguished from uridine by a characteristic fragmentation pattern produced by collision-induced dissociation (CID)-MS (Taucher et al., 2011). Using the same strategy, direct MS-based sequencing of Ψ-containing RNA was used to determine all Ψs in the canonical human spliceosomal snRNAs from 293T cells (Yamauchi et al., 2016). One of the most challenging issues of the LC-MS technique is the identification of RNA modification comparing mass spectra with a sequence database. Implementation of specific programs for database-matching or decoding the complicated patterns of oligonucleotide fragmentation will offer solutions to analyze data from large-scale experiments, allowing the categorization of longer oligonucleotides (Wein et al., 2020).

More recently, using a mass spectrometry-based technology termed SILNAS (stable isotope-labeled ribonucleic acid as an internal standard), all ribosomal modifications of the 80S ribosome from yeast and subsequently from human were mapped (Taoka et al., 2018). This method allowed for a complete site-specific quantitative identification of all RNA modifications exceeding ∼5% in stoichiometry. Unlike other MS-based methods, this approach has the advantage to avoid any molecule modification, however, it requires sophisticated laboratory equipment together with particular expertise. Furthermore, as it necessitates a relatively large amount of RNA (i.e., approximately 5 μg per sample), this MS-based method is not suitable for all RNA classes and is generally not indicated for most scarcely represented transcripts.

The first discovered and most widely exploited method for the study of pseudouridylation was introduced by the use of the chemical reaction of N-cyclohexyl-N′-β-(4-methylmorpholinium) ethylcarbodiimide p-tosylate (CMC) to pseudouridine, which forms a stable adduct blocking reverse transcriptase and hence producing truncated complementary DNA (cDNA) (Bakin and Ofengand, 1998). This method is still used to detect the level of pseudouridine semi-quantitatively at a precise site using fluorescent primer extension and fragment analysis on capillary columns of sequencing apparatus (Deryusheva et al., 2012). An adaptation of the CMC-RT based approach, termed CMC-RT and Ligation Assisted PCR analysis of Ψ modification (CLAP) was recently developed to validate Ψ identification and at the same time quantification of the Ψ modification level at a target site both in ncRNA and mRNA (Zhang et al., 2019). Anyway, this approach can be considered to be at most semiquantitative like other methods exploiting CMC-derivatization, due to either incomplete CMC-Ψ adduct formation and/or a small amount of CMC-Ψ reversion in the CMC procedure.

### High Throughput Methods for Pseudouridine Detection in RNA

High throughput methods are founded on NGS technologies and have been developed in the last decade to achieve a comprehensive view of the pseudouridine landscape in the transcriptome, including *de novo* identification of pseudouridine sites. These approaches combine RNA deep sequencing technologies together with selective chemical derivatization of pseudouridine by CMC. These CMC-based methods, namely Pseudo-Seq (Carlile et al., 2014), Ψ-Seq (Schwartz et al., 2014), PSI-Seq (Lovejoy et al., 2014) and CeU-Seq (Li et al., 2015) adapted the production of truncated cDNA due to the stable adduct formed by CMC to pseudouridine to Illumina massive sequencing. While only slight technical differences reside among library preparation procedures, they differ in the bioinformatic approaches to pseudouridine detection extensively reviewed in (Zaringhalam and Papavasiliou, 2016). By inclusion of synthetic spike-ins and calibrations (Schwartz et al., 2014), deep sequencing CMC-based protocols were made semi-quantitative. These methods were the first to be employed in the transcriptome-wide investigation of pseudouridylation, mainly on mRNA. Nevertheless, they experienced a great variability in the pseudouridylation profile which could be due to chemical limitations in CMC ability to uniformly derivatize pseudouridine and to possible interferences of surrounding modified nucleotides with the Ψ identification. Indeed, CMC-based methods lack sensitivity in Ψ detection on rRNA (for instance, about 50% by Pseudo-seq) (Carlile et al., 2014). Furthermore, as with other indirect NGS-based RNA sequencing techniques, these methods suffer from possible inconsistency between the original RNA molecule and the sequencing products due to the need for cDNA synthesis and PCR amplification. For instance, modifications at the 3′ end of RNA are less frequent due to shorter and less represented fragments after the primer binding sites for reverse transcriptase.

To try to mitigate the tendency of the CMC-based methods to give rise to a significant level of both false positives and false negatives, an alternative technique based on antibody recognition of Ψ residues and UV-crosslinking followed by deep sequencing (photoactivated-Ψ-seq or PA-Ψ-seq) was applied for the study of cellular ncRNAs and mRNAs (Martinez Campos et al., 2021). The PA-Ψ-seq technique showed to efficiently identify pseudouridine modifications present on cellular RNAs but with a sensitivity that in the end is not different from CMC-based methods.

Other sequencing methods have been developed to overcome the many limitations of the CMC-RT approach. The first is based on bisulfite RNA treatment, termed RBS-Seq, which can detect transcriptome-wide at single-base resolution pseudouridine, in addition to m1A and m5C (Khoddami et al., 2019). Regarding Ψ, in presence of heat and magnesium, the bisulfite treatment forms a stable adduct on pseudouridine leading to ribose ring-opening and reorientation. This forces the reverse transcriptase to skip the base, leaving a deletion signature at the exact modified sites. With this technique, quantitative and simultaneous detection of three important modifications was achieved in the same sample, in coding and non-coding RNAs, even at low abundance. RBS-Seq has the advantage of providing a high throughput validation protocol for Ψ sites easily adapted to mRNA without the need for large quantities of pure target RNA. Furthermore, as this method does not stop reverse transcriptase, it can distinctly recognize two contiguous Ψ sites on the same RNA. Despite the harsh conditions of bisulfite treatment, the use of this technique made it possible to detect modifications also in the short 5.8S rRNA. Nevertheless, the target application of this approach is not represented by rRNA due to its characteristic abundance of different modifications in close proximity which could interfere with Ψ detection. As a matter of fact, by RBS-Seq, not all the 104 pseudouridine modifications harbored on the 80S ribosome were identified (for instance only 74 in HeLa cell lines).

The second CMC-alternative high throughput method relies on RNA cleavage at uridine residues by hydrazine, followed by aniline treatment, and resistance of pseudouridine to chemical treatment (Marchand et al., 2020). This method termed HydraPsiSeq was applied for precise mapping and quantification of pseudouridine residues in yeast and human rRNAs and tRNAs and was revealed to provide highly sensitive, reliable and precise quantification of Ψ in any particular position in RNA. Analysis of scarce transcripts, such as mRNAs is also feasible but only by highly increasing the sequencing depth (for instance to 100 million for yeast mRNA) and therefore the expected cost. So, for this purpose, an enrichment step with a Ψ-specific antibody is suggested (Slama et al., 2019).

All the described techniques have the great drawback of modifying RNA molecules and using customized protocols to map each specific modification individually. Furthermore, cluster sequencing involves an amplification step, experiencing potential bias and providing only an average picture of modifications in a population of RNA molecules (for a detailed update in Second- and Third-Generation Deep Sequencing of RNA modifications see Motorin and Marchand, 2021).

Promising results would be expected from single-molecule sequencing approaches, also called third-generation sequencing. They are based on two independent principles: single-molecule real-time (SMRT) technology using nano wells (called zero-mode waveguides, ZMWs, Pacific BioSciences) and nanopore sequencing (Min-ION, Oxford Nanopore Technologies) (Schwartz and Motorin, 2017). The main advantage of both technologies is their very long read length >10,000 nt on average and therefore the possibility to sequence full-length native RNA molecules in principle providing the possibility to map the individual modification pattern along the same RNA molecule without the need of aligning short reads. Nanopore sequencer use flow cells containing an array of nanopores embedded in an electro-resistant membrane. Each nanopore corresponds to its own electrode connected to a channel and sensor chip, which measures the electric current that flows through the nanopore. The passage of the nucleic acid through a nanopore disrupts the current producing a characteristic ‘squiggle’. The squiggle is then decoded using base-calling algorithms to determine the DNA or RNA sequence in real time. Nanopore sequencing can detect modified bases according to differences in squiggles between modified and unmodified base. Recent advances have been made in the detection of base modifications using the Nanopore sequencer (Xu and Seki, 2020), including direct sequencing of RNA (Garalde et al., 2018; Stephenson et al., 2022). By the new application methods and more accurate bioinformatic tools, nucleotide modifications in *E. coli* 16S rRNA (Smith et al., 2019) and tRNA (Thomas et al., 2021) were detected. Great efforts are made to implement tools for the detection of RNA modifications, including machine learning classifiers (neural network, random forest, logistic regression, and naive Bayes classifiers) or statistical test-based tools which can detect *de novo* modifications without training using modified and unmodified samples (Xu and Seki, 2020). Among the latest bioinformatic tools, promising appear to be ELIGOS (epitranscriptional landscape inferring from glitches of ONT signals) (Jenjaroenpun et al., 2021) NanoPsu (Huang et al., 2021) and Penguin (Hassan et al., 2022). However, at the moment the overall precision of pseudouridine detection with this sequencing method remains low and not quantitative (Begik et al., 2021) for native full-length molecules extensively modified such as human rRNA.

Finally, single-particle cryo-electron microscopy (cryo-EM) is a rapidly developing technique, which already depicted the structure of the 80S ribosome, together with rRNA modifications at an average of 3Å resolution (Natchiar et al., 2017; Natchiar et al., 2018). By this approach, it was possible to show that the majority of modifications reside in domains crucial for translational activity which are conserved from bacteria to superior eukaryotes, where ribosomes acquired an extended “shell of modification” which fine-tune protein synthesis (Natchiar et al., 2017). However, numerous base inconsistencies have been recognized so far between different approaches (Taoka et al., 2018). The use of even more advanced algorithms and software (Kimanius et al., 2021), together with the refinement of the technique itself to achieve higher resolution (Pellegrino et al., 2022), will provide fundamental information on all the ribosomal modifications and detailed interactions in the three-dimensional structure of the native human ribosome. Although some limitations still exist due to the low throughput and time-consuming techniques, cryo-EM will be extremely useful to recognize how particular molecules can bind to specific modified sites in ribosomes and inhibit their activity, offering new insights for structure-based drug design (Gilles et al., 2020; Poitevin et al., 2020).

## Discussion and Perspectives

Given these recent developments, identifying the modifications in the chemical pattern of rRNA may well represent a new path towards formulating a true picture of the functional specialization of ribosomes. As described above, ribosome function and activity can be impacted by the composition in ribosomal proteins and the plasticity in rRNA which encompasses diversity in modifications and interaction with certain factors. Any changes in ribosomal function or activity may affect the regulation of gene expression in both normal and abnormal conditions. Although the exact mechanism through which an altered or oncogenic ribosome may affect tumor progression is not yet fully defined, a correlation between anomalies in ribosome biogenesis or function and cancer has been reported. This has helped in defining the link between alterations in translation apparatus and cancer etiology.

As previously stated pseudouridylation is one of the most abundant chemical modifications affecting ribosome function. In general, these modifications can be driven by either stand-alone pseudouridine synthases or snoRNA H/ACA box guided pseudouridylation complexes. Pseudouridine synthases are classified into six families and are known to recognize the substrate and catalyze the conversion of uridine to pseudouridine without using any cofactors.

On the other hand, RNA dependent pseudouridylation is carried out by H/ACA box snoRNA. H/ACA box snoRNAs, also known as SNORAs (present in all eukaryotes and in archaea as sno-likeRNA as well (Czekay and Kothe, 2021)), possess a two hairpin structures connected by a hinge region bearing a conserved (ANANNA) motif (also known as the H motif, BOX H) and an ACA box motif at their 3′end (Balakin et al., 1996). Pseudouridylation in rRNA is induced by SNORA molecules after forming a complex with a set of proteins. The core proteins involved are dyskerin (orthologues Cbf5 in yeast, Nap57 in rat and NOP60B in Drosophila), non-histone protein 2 (NHP2), nucleolar protein 10 (NOP10) and glycine-arginine-rich protein 1 (GAR1). As mentioned above, dyskerin is known to exhibit enzymatic activity in this complex. Moreover, it has been proved that epitranscriptome activity of rRNA is affected by dysregulation of dyskerin (Janin et al., 2020).

In numerous studies snoRNAs have also been directly related to cancer initiation and progression. Schulten and others explored the snoRNA expression in patients with breast cancer brain metastasis in comparison to non-metastatic breast cancers. The study revealed that 13 SNORAs were differentially expressed and possibly involved in supporting metastatic progression (Schulten et al., 2017). Similarly, another research group identified 21 snoRNA to be correlated with metastasis in prostate cancer. The study highlighted SNORA55 to be a possible driver gene in prostate cancer (Crea et al., 2016). Moreover, in an independent study on non-small cell lung cancers (NSCLC) researchers developed a prediction model based on SNORA78, SNORA68 and SNORA47 for early detection and prognostication of NSCLC (Gao et al., 2015). In another study, SNORA42 was found to be associated with invasiveness and metastasis of non-small lung carcinoma by regulating the stemness of lung CSC. The decreased expression of SNORA42 was linked to a decrease in self-renewal ability and suppression of tumor initiation in xenografted mice (Mannoor et al., 2014). Therefore, it is safe to say that the growing evidence leaves no doubt regarding the role of SNORA in cancer development and their potential use as biomarkers for diagnosis, prognosis and potential treatment.

However, despite the growing link of snoRNA with disease prognosis and progression very little is known about the method of action and the pathways adopted by these molecules to carry out the effect. Increased levels of DKC1 and H/ACA snoRNA together with elevated levels of pseudouridine were reported *in vitro* studies conducted on prostate cancer cells (Uddin et al., 2020). Another study reported a similar connection between the elevated expression of DKC1 with upregulated expression of SNORA67 and increased U1445 modification on 18S ribosomal RNA in a breast cancer cell line. They also reported high levels of DKC1 associated with certain tumoral features (Guerrieri et al., 2020). This depicts a possible link between pseudouridine levels and snoRNA with cancer initiation, progression and metastasis. Accordingly, in a recent study, McMahon and others characterized the role of SNORA24 in hepatocellular carcinoma and its cooperation with oncogenic RAS mutations. The modification of uridine into pseudouridine is driven by SNORA24 at positions 609 and 863 on 18S rRNA. Ribosomes missing these modifications fail to exhibit the correct selection of aminoacyl-transfer RNA and ribosome complex formation pre-translocation (McMahon et al., 2019).

It would be worth mentioning here that the ribosomes can be targeted using drugs with different active molecules (Gilles et al., 2020). Consequently, knowing the elements that take part in causing the unusual level of modifications resulting in the development of an abnormality i.e. oncoribosomes can be exploited in devising the treatment.

**FIGURE 1:**
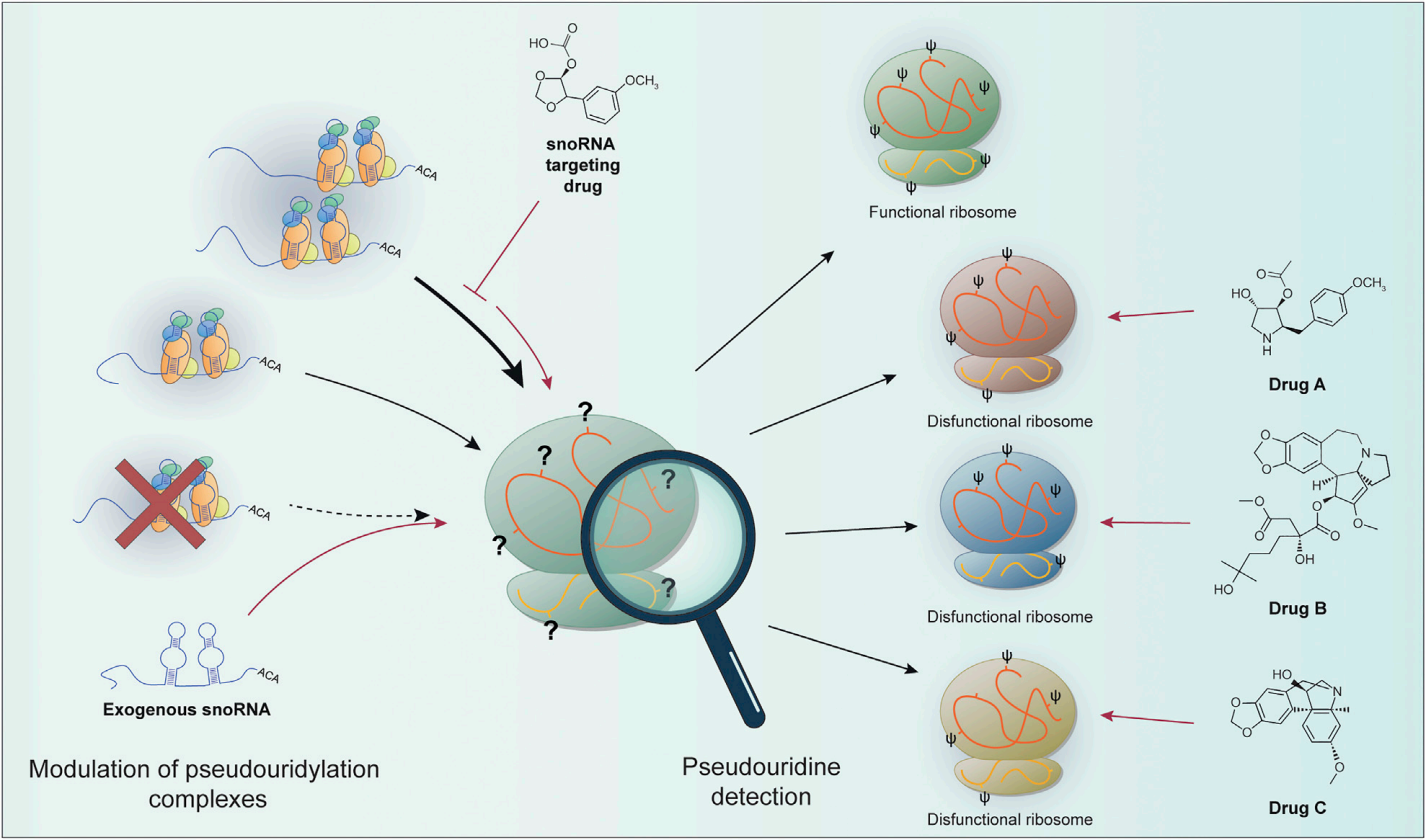
The levels of site-specific pseudouridylation can be affected by alterations in the components of the pseudouridylation complex (e.g., re-modulation on snoRNA expression or pseudouridine core proteins impairment). snoRNA-based targeted therapies (e.g., snoRNA targeting drugs or exogenous snoRNA sequences) can in principle restore proper rRNA modification. Site-specific pseudouridine detection can allow the identification of particular ribosomal populations. These features can be exploited to develop drugs targeting specific ribosomal positions containing dysregulated pseudouridine sites. It could be then possible to take advantage of this peculiarity in cancer to target only the cells with modified ribosomes, the so called “oncoribosomes”. Prospectively, the characterization of ribosome modifications (or snoRNA expression as a surrogate) can be used to specifically target cancer cells.

To sum up, we can expect that the recent advancement in the techniques that could identify the chemical modifications in rRNA molecules reported here could in perspective take us one step forward in better understanding the role played at the molecular level by altered rRNA pseudouridylation in disease development and sequentially in the development of strategies for better disease management and the development of targeted therapies snoRNA- or ribosome-centered (Figure 1).
